# Supplementary material for: Toward simple, rapid, and deep plant proteome analysis with an in-cell proteomics strategy
Source: bioRxiv. 2025 Oct 31:2025.10.30.685699. Preprint. [Version 1] doi: 10.1101/2025.10.30.685699 (PMC12636300; doi:10.1101/2025.10.30.685699)
Supplement: Supplement 1 [file NIHPP2025.10.30.685699v1-supplement-1.pdf]

bioRxiv preprint doi: https://doi.org/10.1101/2025.10.30.685699; this version posted October 31, 2025. The copyright holder for this preprint (which was not certified by peer review) is the author/funder, who has granted bioRxiv a license to display the preprint in perpetuity. It is made available under aCC-BY 4.0 International license.

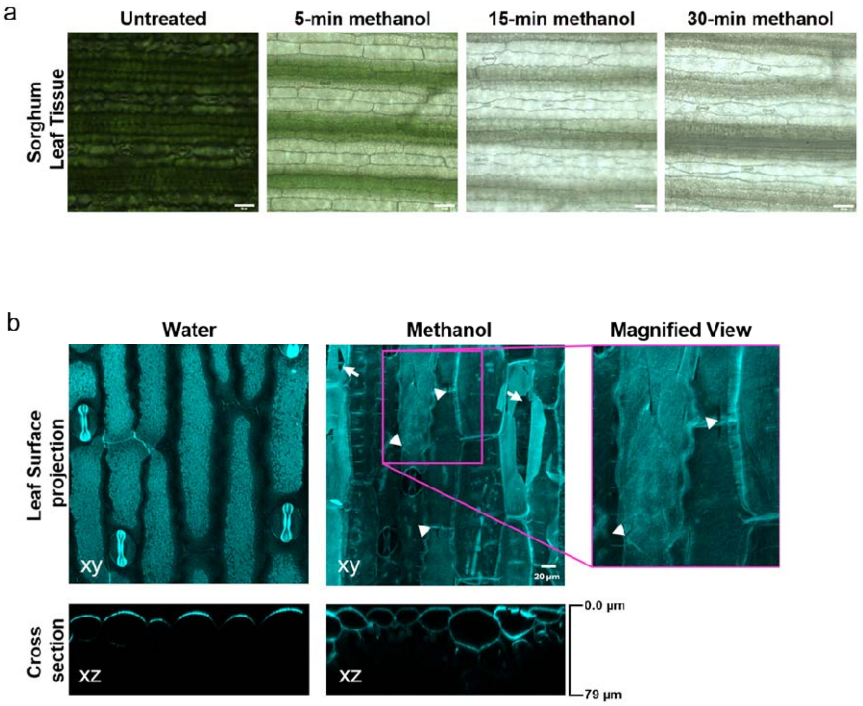

Supplementary Figure S1. Microscopic assessment of sorghum leaf upon fixation. (**a**) Brightfield images showing the effect of 0, 5, 15, and 30 minutes of methanol treatment on leaves of *Sorghum bicolor*. Scale bar equals 50 µm. (**b**) Sorghum leaf sections treated with water or methanol, labeled with the cell wall dye Calcofluor White MR, and then imaged using multiphoton microscopy. Maximum intensity projections of the leaf surface (XY) show small cracks along the cell wall junctions (arrowheads) and larger breaks in the cell walls (arrow). A cross-sectional view (XY) comparing the staining and imaging depth between water and methanol cleared samples. Scale bar equals 20 µm.

bioRxiv preprint doi: https://doi.org/10.1101/2025.10.30.685699; this version posted October 31, 2025. The copyright holder for this preprint (which was not certified by peer review) is the author/funder, who has granted bioRxiv a license to display the preprint in perpetuity. It is made available under aCC-BY 4.0 International license.

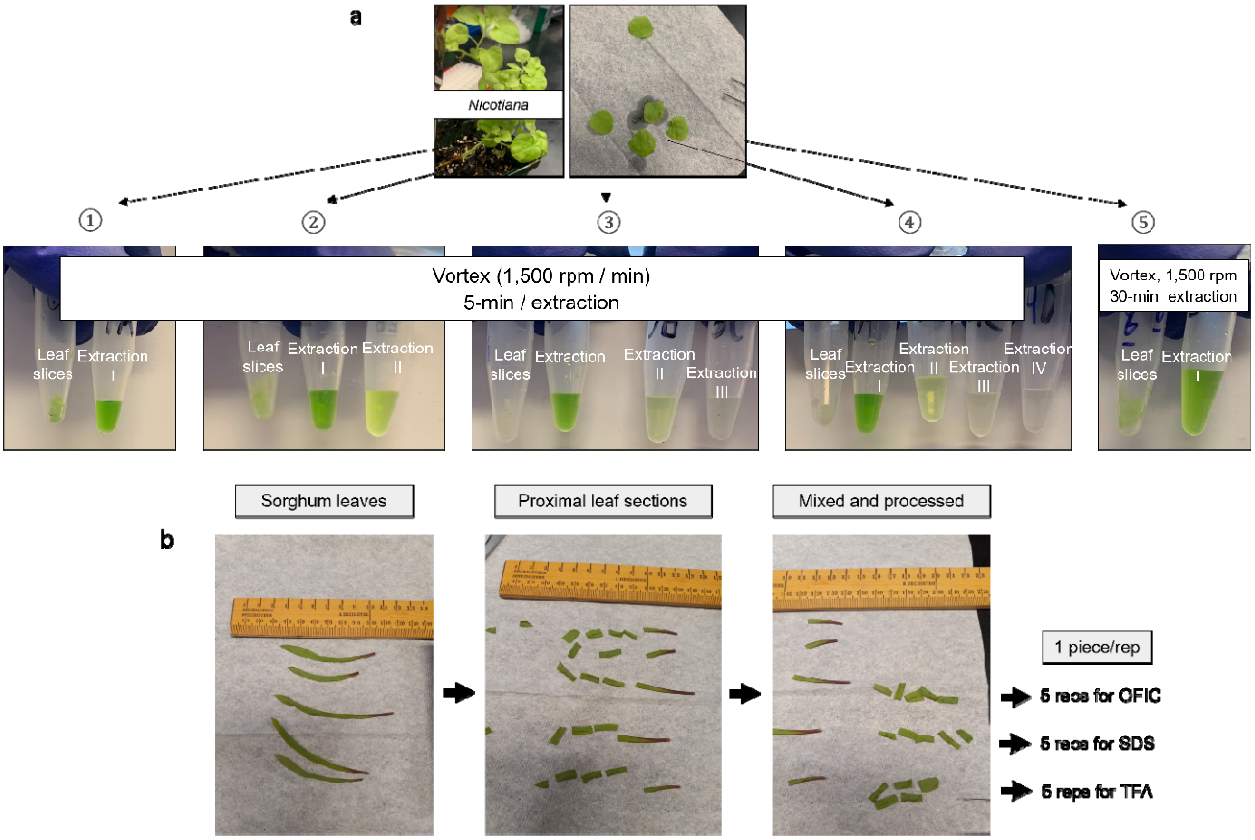

Supplementary Figure S2. Examination of methanol fixation and leaf sample preparation. **(a)** *Nicotiana benthamiana* leaves after clearing with 1, 2, 3, or 4 exchanges (①-④) of methanol compared to a single 30-minute methanol treatment (⑤). **(b)** *Sorghum* leaf sample processing for proteomics analysis.

bioRxiv preprint doi: https://doi.org/10.1101/2025.10.30.685699; this version posted October 31, 2025. The copyright holder for this preprint (which was not certified by peer review) is the author/funder, who has granted bioRxiv a license to display the preprint in perpetuity. It is made available under aCC-BY 4.0 International license.

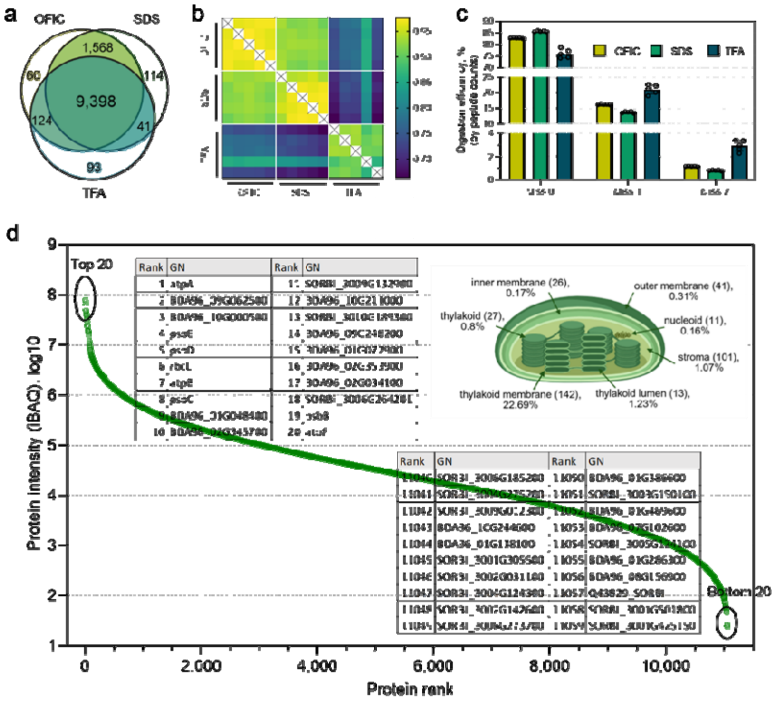

Supplementary Figure S3. Evaluation of OFIC digestion for sorghum leaf proteomics. **(a)** Venn diagram of the overall proteins identified by each processing method. **(b)** A Pearson's correlation analysis between replicates and treatments. **(c)** Comparison of the digestion efficiency of OFIC, SDS, and TFA methods as represented by the percentage of peptides with 0, 1, or 2 missed cleavages. (**d**) Dynamic range of sorghum leaf proteome. The 20 most and least abundant proteins (top 20 and bottom 20) are indicated on the plot. Inner panel depicts the chloroplast proteins and their relative abundance. Numbers in the brackets indicate the number of proteins of each category.

bioRxiv preprint doi: https://doi.org/10.1101/2025.10.30.685699; this version posted October 31, 2025. The copyright holder for this preprint (which was not certified by peer review) is the author/funder, who has granted bioRxiv a license to display the preprint in perpetuity. It is made available under aCC-BY 4.0 International license.

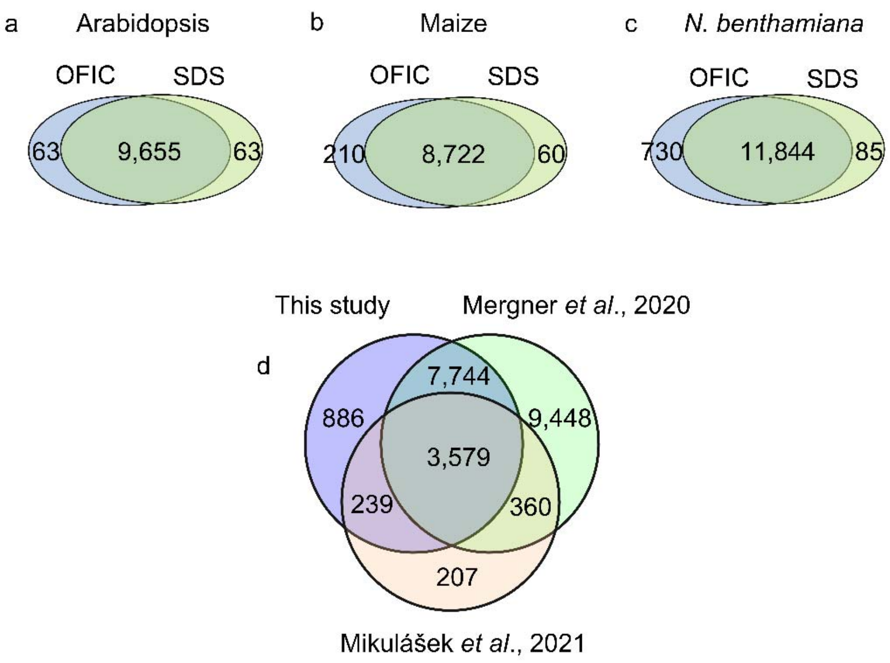

Supplementary Figure S4. Venn diagram analysis. **(a-c)** Comparisons of protein identifications derived from OFIC and SDS digestion methods for Arabidopsis, maize, and N. benthamiana leaves, respectively. **(d)** Overlapping analysis of Arabidopsis proteins identified by this study (in-cell digestion and single-shot LCMS) with two other studies by Mergner et al., 2020 (homogenization and urea-based in-solution digestion, high pH fractionation, and multi-shot LCMS), and by Mikulášek et al. 2021 (SDS lysate and single-shot LCMS). Please be noted that the numbers used here are slightly different from the numbers reported in the main text in this study and the Mergner's. We used all the protein accessions within each protein groups, if there are multiple members in one group, to maximize the overlaps between different studies.

bioRxiv preprint doi: https://doi.org/10.1101/2025.10.30.685699; this version posted October 31, 2025. The copyright holder for this preprint (which was not certified by peer review) is the author/funder, who has granted bioRxiv a license to display the preprint in perpetuity. It is made available under aCC-BY 4.0 International license.

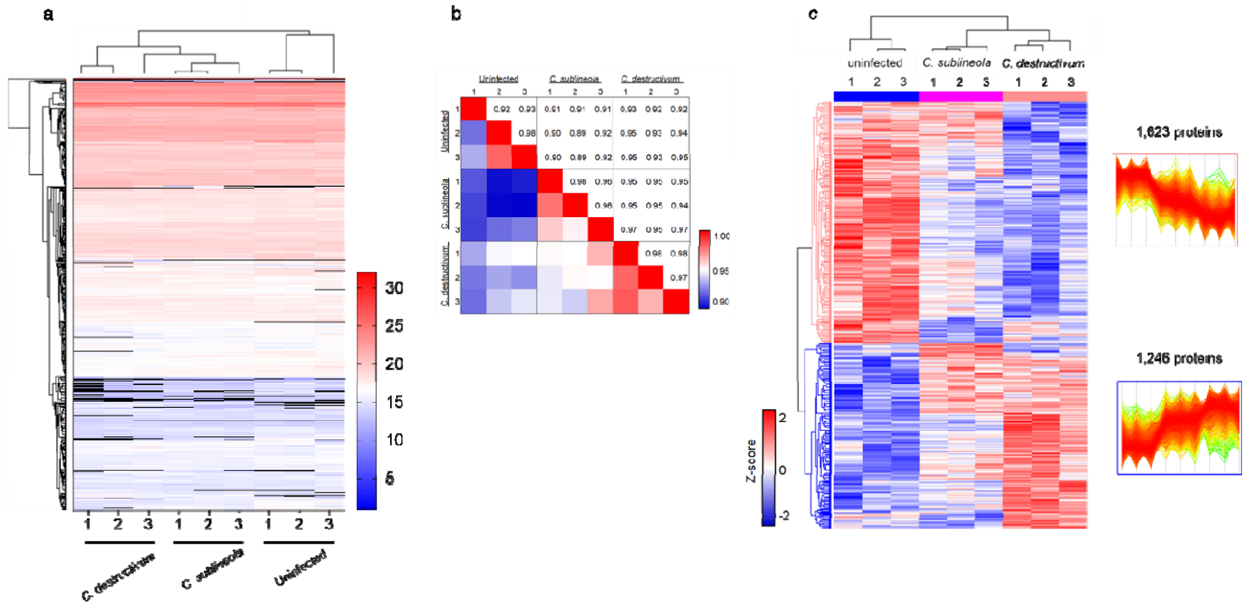

Supplementary Figure S5. Quantitative analyses of plant leaf infection. (**a**) Unsupervised hierarchical clustering analysis of the three groups. Dark lines indicate the proteins that have missing values. (**b**) Heatmap of Pearson correlation. (**c**) Heatmap of ANOVA significant proteins among the three groups. Significance cutoff is $p < 0.05$. The number of proteins in the two clusters (pink and blue highlighted) are depicted.

bioRxiv preprint doi: https://doi.org/10.1101/2025.10.30.685699; this version posted October 31, 2025. The copyright holder for this preprint (which was not certified by peer review) is the author/funder, who has granted bioRxiv a license to display the preprint in perpetuity. It is made available under aCC-BY 4.0 International license.

In-cell proteomics analysis of plant leaf using E3filter

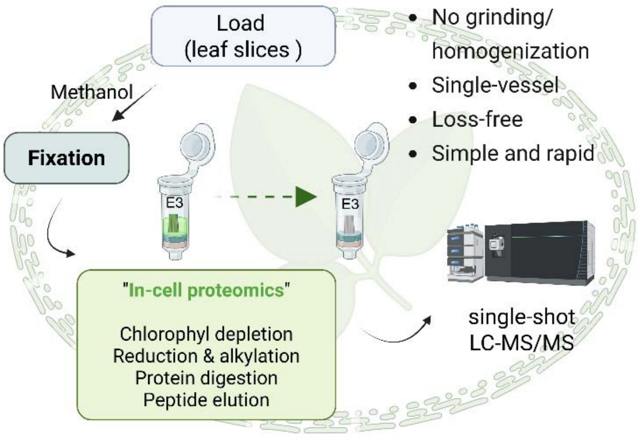

1. **Sample pretreatment and loading**
   Collect leaves from plants; a 1-2 cm^2 section would be sufficient for one digestion experiment. Further slice it into 1-2 mm^2 pieces, and transfer them to E3filter (with a tweezer).

2. **Methanol fixation and chlorophyl depletion**
   Add 200-400 µl methanol, shake at 1,000 rpm for 5 min under room temperature. Centrifuge at 3,000 rpm for 1 min, discard flow through. Repeat this step (e.g.,3-6 times) until the flow through is clear with no green color.

3. **Reduction and alkylation**
   Depending on the sample size, add 100-200 µl of 50 mM triethylammonium bicarbonate (TEAB) to cover the leaf pieces, and final concentration of 10 mM Tris(2-carboxyethyl)phosphine (TCEP), incubate at 45°C for 10-15 min with gentle shaking (300-500 rpm).

4. **Wash**
   Centrifuge at 3,000 rpm for 1 min to eliminate liquid. Add 200 µl of 50 mM TEAB solution, centrifuge again, and discard flow through.

5. **Digestion**
   Transfer E3filters to clean collection tubes (here, 2-ml tube is suggested), add 150 µl of 50 mM TEAB, desired enzyme (Trypsin or Trypsin/Lys-C mix) at 1:50 ratio. Incubate at 37°C for 16-18 hours with gentle shaking. For 1-cm^2, 1 µg of enzyme is suggested.

6. **Elution**
   After digestion, spin filters at 3,000 rpm for 1 min to collect flow through. Do two additional elutions by adding 150 µl of 0.2% formic acid in water, and 150 µl of 0.2% formic acid in 50% acetonitrile, respectively, spin and collect flow through to the same collection tube.

7. **Drying and desalting**
   Dry digests in SpeedVac; do desalting following standard StageTip protocol.

bioRxiv preprint doi: https://doi.org/10.1101/2025.10.30.685699; this version posted October 31, 2025. The copyright holder for this preprint (which was not certified by peer review) is the author/funder, who has granted bioRxiv a license to display the preprint in perpetuity. It is made available under aCC-BY 4.0 International license.

In-cell proteomics analysis of plant pollen using E4tip

1. **Sample collection and loading**
   Collect pollen grains, aliquot 20-50 µl to E4tips that are prefilled with 150-200 µl of pure methanol.
2. **Fixation**
   Spin tips at 4,000 rpm for 2 min, discard flow through. Add 200 µl methanol to samples, and incubate at room temperature for 15 min. Centrifuge at 4,000 rpm for 2 min, discard flow through. Tip: the flow through may be collected here for metabolomics analysis.

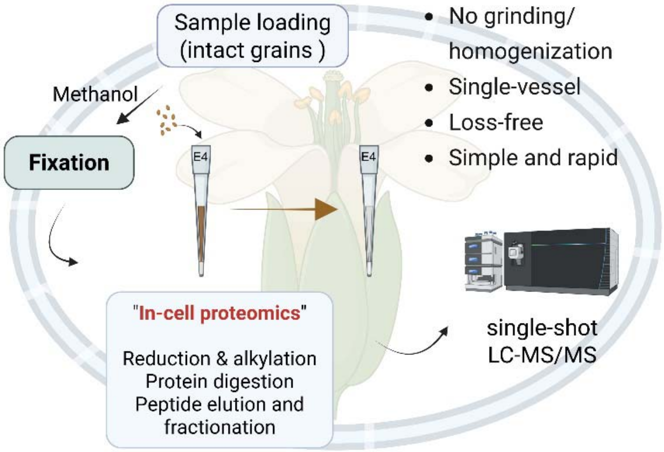

3. **Reduction and alkylation**
   Add final concentration of 10 mM Tris(2-carboxyethyl)phosphine (TCEP) and 40mM chloroacetamide (CAA) in 100 µl of 50 mM triethylammonium bicarbonate (TEAB), incubate at 45°C for 10-15 min with gentle shaking (300-500 rpm).
4. **Wash**
   Centrifuge at 4,000 rpm for 1 min to eliminate liquid. Add 200 µl of 50 mM TEAB solution, centrifuge again, and discard flow through.
5. **Digestion**
   Transfer E4tips to clean collection tubes, add 150 µl 50 mM TEAB, desired enzyme (Trypsin or Trypsin/Lys-C mix) at 1:50 ratio. Incubate at 37°C for 16-18 hours with gentle shaking.
   Tip 1: please make sure no air gap between the buffer and the membrane filter. Do a pulse spin if desire (i.e., 2,000 rpm for 2 seconds).
   Tip2: No caps are required for E4tips during overnight incubation.
6. **Acidification and desalting**
   After digestion, add formic acid to final concentration of 1%, centrifuge at 1,500 rpm for 10 min. Add 200 µl 0.5% acetic acid in water, centrifuge at 4,000 rpm for 2 min, discard flow through.
7. **Elution**
   Transfer E4tips to clean collection tubes, do two sequential elution by adding 200 µl 60% acetonitrile/0.5% acetic acid in water (elution I), and 80% acetonitrile/0.5% acetic acid in water (elution II); centrifuge at 4,000 rpm for 2 min to collect eluants to the same tube. Dry samples in the SpeedVac, and store at -80°C. The peptides are now desalted and ready for LCMS analysis.
